# Thrombin is a therapeutic target for non-small-cell lung cancer to inhibit vasculogenic mimicry formation

**DOI:** 10.1038/s41392-020-0167-1

**Published:** 2020-07-10

**Authors:** Bing Zhao, Mengfang Wu, Zhihuang Hu, Yixin Ma, Wang Qi, Yanling Zhang, Yaran Li, Min Yu, Huijie Wang, Wei Mo

**Affiliations:** 1 Key Laboratory of Metabolism and Molecular Medicine, Ministry of Education, Fudan University, Shanghai, China; 2 The Department of Biochemistry and Molecular Biology, School of Basic Medical Sciences, Fudan University, Shanghai, China; 3 Department of Oncology, Shanghai Medical College, Fudan University, Shanghai, China; 4 Department of Medical Oncology, Shanghai Cancer Center, Fudan University, Shanghai, China; 5 Collaborative Innovation Center for Biotherapy, Sichuan University, Chengdu, China

**Keywords:** Drug development, Target identification

## Abstract

Tumor cells transform into endothelial cells by epithelial-to-mesenchymal transition, which is characterized by vasculogenic mimicry (VM). VM not only accelerates tumor progression but also increases drug-induced resistance. However, very little is currently known about the molecular determinants that enable VM. Targeting VM might bring a new breakthrough in cancer treatment. Thrombin is the key enzyme of the blood coagulation system and could contribute to tumor progression. Nevertheless, the association between thrombin and VM formation remains largely unknown. We found that VM was associated with the overall survival of non-small-cell lung cancer (NSCLC) patients, and that thrombin expression was closely related to VM formation. This research revealed that thrombin induced VM formation via PAR-1-mediated NF-κB signaling cascades. The novel thrombin inhibitors r-hirudin and DTIP inhibited VM formation and spontaneous metastases in subcutaneous tumors. Clinical pathological analysis confirmed that NSCLC patients with thrombin-positive/PAR-1-high expression had the poorest prognosis and were the most likely to form VM. The promotional activity of thrombin in VM formation and tumor metastasis was abolished in PAR-1-deficient NSCLC cells. The EGFR inhibitor gefitinib had no effect on VM and increased VEGF expression in tumors. The combination therapy of DTIP and gefitinib achieved a better therapeutic effect than either agent alone. This study is the first to illustrate that thrombin substantially contributes, together with PAR-1, to VM formation and to illustrate that VM might be a target of r-hirudin and DTIP to suppress tumor progression. The anticoagulants r-hirudin and DTIP could be employed for antitumor therapy. Combination therapy with DTIP with an EGFR inhibitor might achieve superior therapeutic effects.

## Introduction

Lung cancer is one of the deadliest types of cancer in the world^1^ and approximately 80–85% of lung cancers are non-small-cell lung cancers (NSCLCs). Despite many advances in the treatment of NSCLC in recent years, the median survival is still <12 months and <15% of NSCLC patients survive more than 5 years from the initial diagnosis.^2^ Even if NSCLC patients are treated with chemotherapy or radiotherapy, the incidence of recurrence is still high.^3,4^ Compared with traditional chemotherapy, precise tumor treatment is helpful to improve the treatment effect and quality of life. Targeted therapy has become an important means of disease treatment for NSCLC patients.^5^

The growth, invasion, and metastasis of tumors cannot be separated from the new blood vessel supply of additional oxygen and nutrients.^6^ Tumor angiogenesis is the key condition for tumor growth and metastasis, so antiangiogenic treatment is currently one of the main treatment strategies for lung cancer. Angiogenesis inhibitors, such as epidermal growth factor receptor (EGFR) inhibitors, have been used to limit tumor growth;^7^ however, traditional angiogenesis inhibitors are not ideal in solid tumor treatment, which suggests that there may be another relatively independent microvascular system involved in tumor blood supply. In recent years, tyrosine kinase inhibitors (EGFR-TKIs) have been used in NSCLC patients.^8^ Gefitinib, an EGFR-TKI, is used to treat advanced NSCLC patients. Despite the significant response to gefitinib, these tumors always develop resistance within an average of 9–12 months.^9^

In 1999, Maniotis et al.^10^ found that cancer cells cover nonendothelial vascular channels containing red blood cells, which is called vasculogenic mimicry (VM). The formation of VM not only accelerates tumor metastasis but also increases the risk of resistance to antiangiogenic therapy in NSCLC.^11^ In addition, it is an interesting possibility that antiangiogenic therapy may lead to the formation of VM, thus allowing drug-induced resistance to occur.^12^ However, current antiangiogenic drugs are mainly based on the inhibition of endothelial cell-dependent angiogenesis, ignoring the existence of VM. Therefore, targeting the formation of VM may bring breakthroughs in cancer treatment.

The transcriptional signature of VM^13^ shares components with signatures of “stemness” and epithelial-to-mesenchymal transition (EMT), key attributes related to tumor plasticity during metastasis and drug-induced resistance.^14–16^ A series of studies have confirmed that EMT is involved in the formation of VM.^16^ Epithelial endothelial transition, a subtype of EMT, produces endothelial-like phenotype of tumor cells, whereas endothelial-like phenotype can form VM to allow serum into tumor tissues.^17^ Serum contains prothrombin, which is converted into thrombin via proteolytic cleavage in the tumor microenvironment.^18^ However, the level of thrombin in NSCLC tumor tissues and the relationship between thrombin and VM are still unclear.

It has long been presumed that tumors may take advantage of the hemostatic system. A relationship between increased clotting and malignancy was recognized more than a century ago. Thrombin exerts a variety of biological effects by interacting with various receptors on the surface of vascular and nonvascular cells.^19^ Studies have shown that thrombin plays important roles in tumorigenesis, inflammation, and metastatic dissemination of tumor cells through its PAR-1 receptor.^20–22^ Thrombin binds to the hydrophobic hirudin-like sequence DK^51^YEPF^55^ on the PAR-1 extracellular face via exosite I, facilitating the interaction of the PAR-1 cleavage site with the active site of thrombin. Thrombin cleaves the N-terminal extracellular domain of PAR-1 and the newly exposed N terminus interacts with the second extracellular domain of the cleaved receptor to initiate signaling. The cleavage of PAR-1 by thrombin initiates potent inflammatory responses, including the upregulation of cell surface adhesion molecules and the induction of hyperpermeability.^23^ It was reported that thrombin, acting through PAR-1, promotes EMT in embryo development.^24,25^ However, the relationship between thrombin, PAR-1, and VM in human NSCLC has not been reported thus far; we suppose that thrombin may induce VM through PAR-1 activation.

Direct thrombin inhibitor peptide (DTIP) and recombinant hirudin (r-hirudin), which are derivatives of wild-type hirudin variant 2, were developed by our group.^26,27^ DTIP and r-hirudin bind to exosite I and to the apolar region of thrombin, whereas the N-terminal moiety of r-hirudin and DTIP blocks access to the thrombin active site, to inhibit the activity of thrombin. r-hirudin has entered phase I clinical trials and DTIP is a novel antithrombotic agent that can be used to prevent thrombosis without conferring an increased bleeding risk for subcutaneous (s.c.) injection. In our recent studies, we found that r-hirudin and DTIP could suppress progression, dissemination, and spontaneous metastasis in NSCLC.

Therefore, in this study, we first investigated the levels of thrombin in clinical NSCLC samples and explored the relationship between thrombin level and VM formation. We evaluated the effects of r-hirudin and DTIP on VM formation in vitro and in vivo. We first examined the potential roles of thrombin and PAR-1 in regulating the formation of VM in NSCLC. We evaluated the effects of combination therapy with DTIP and gefitinib in NSCLC. Thrombin may be a potential target for future anticancer therapies and combination therapy with direct thrombin inhibitors and EGFR inhibitors might achieve a superior therapeutic effect.

## Results

### The formation of VM in NSCLC is closely related to the level of thrombin and the prognosis of patients

**Fig. 1:**
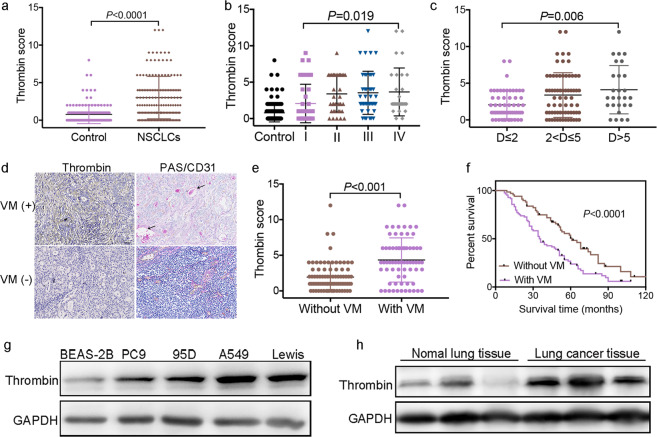
Thrombin is overexpressed in NSCLC and the formation of VM in NSCLC is closely related to the level of thrombin and the prognosis of patients. **a** Score of thrombin expression in adjacent nontumor lung tissue and in NSCLC tissue. **b** Thrombin expression in different stages (AJCC Tumor-node-metastasis (TNM) stages) of NSCLC. **c** The association of thrombin expression with tumor size in NSCLC samples, where D is the maximum tumor diameter. **d** Region showing typical malignant morphology of NSCLC and thrombin expression in corresponding tissues. PAS+/CD31− VM tubes (black arrow) are also shown. **e** The relationship between the expression level of thrombin and the formation of VM in tumor tissues. **f** Univariate survival analysis according to VM in NSCLC patients and Kaplan–Meier survival analysis for patients dichotomized by the presence VM. **g** The expression of thrombin in BEAS-2B, PC9, 95D, A549, and Lewis cells was determined by western blotting. **h** The expression of thrombin in tumor tissues of mice and normal lung tissues of mice was determined by western blotting. All the results are expressed as the mean ± SD. The error bars indicate the SD. ANOVA followed by Dunnett’s test was applied for multiple comparisons. *p < 0.05

VM is detected in clinically by CD31/periodic acid schiff (PAS) double-staining of PAS positive, CD31 negative vessels.^11,14,28,29^ We analyzed the expression levels of thrombin and the formation of VM in 152 NSCLC cases against detailed clinical and pathologic information. We found that the thrombin score was higher in NSCLC tissues (3.12 ± 2.82) than in adjacent nontumor lung tissues (0.77 ± 1.13, Fig. 1a) and upregulated thrombin expression was observed in 126 of 152 human NSCLC tumor samples (82.9%). Furthermore, thrombin expression in tumor tissue was correlated with clinicopathological features, such as tumor-node-metastasis (TNM) stage (Fig. 1b) and tumor size (Fig. 1c). Thrombin expression in patients in phase III (3.69 ± 3.29) or phase IV (3.91 ± 3.75) was significantly increased compared with that in patients in phase I (1.96 ± 2.30) and thrombin expression was higher in patients with a maximum tumor diameter > 5 cm (4.28 ± 3.29) than in patients with a maximum tumor diameter ≤ 2 cm (2.05 ± 1.90). Tissues were stained with PAS and anti-CD31 antibodies to investigate VM. Typical VM structures were found in 68 (68/152) lung cancer specimens. Univariate survival analysis demonstrated that patients with VM (35.4 ± 4.8 months) had a reduced 16-month survival compared with patients without tumor VM (51.2 ± 5.2 months, Fig. 1f). Patients with VM (20.0 ± 3.5 months) had a worse prognosis and were more likely to relapse after surgery than patients without VM (38.0 ± 5.3 months, Supplementary Fig. S2a). Importantly, compared with patients without VM (1.93 ± 1.86), patients with VM (4.4 ± 3.09) had a high expression of thrombin in lung tumors (Fig. 1d, e and Supplementary Fig. S1). Moreover, compared with that in a normal lung cell line (BEAS-2B), thrombin expression in four NSCLC cell lines was significantly increased (Fig. 1g and Supplementary Fig. S2b). The expression of thrombin in tumor tissues of mice was also increased compared with that in paired normal lung tissues (Fig. 1h and Supplementary Fig. S2c).

### r-hirudin and DTIP inhibit thrombin-promoted VM formation in vitro by inhibiting EMT

**Fig. 2:**
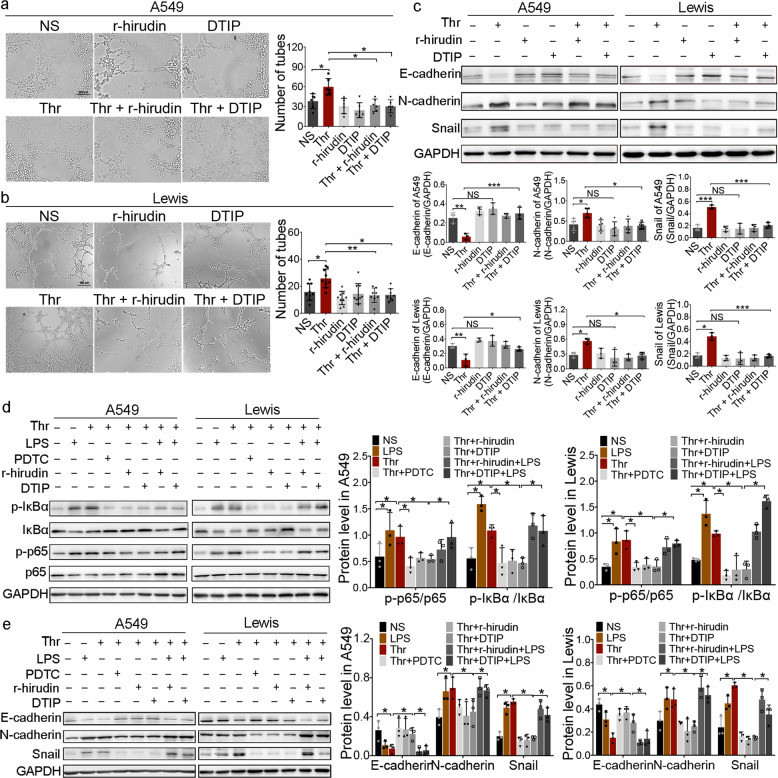
r-hirudin and DTIP inhibit VM formation of A549 and Lewis cells in vitro. **a**, **b** A549 and Lewis cells were pretreated with PBS, 10 nmol/L thrombin, 25 nmol/L r-hirudin, 50 nmol/L DTIP, 10 nmol/L thrombin+25 nmol/L r-hirudin, or 10 nmol/L thrombin+50 nmol/L DTIP, and VM-like network formation on Matrigel in different groups was examined. Left, representative photographs of experiments. Right, quantification of the inhibitory activity of r-hirudin and DTIP on VM tube formation. **c** The effect of thrombin, r-hirudin and DTIP on the expression of snail and other EMT markers was determined by western blotting analysis. GAPDH was used as the loading control. The summarized western blot data for EMT markers are given (bottom). **d** Different groups were probed for phosphorylated p65, phosphorylated IκBα and total p65 and IκBα. GAPDH was used as the loading control. Right, summarized western blot data. **e** The expression of snail and other EMT markers in different groups was determined by western blotting analysis. Right, the summarized western blot data for EMT markers. All the results are expressed as the mean ± SD. The error bars indicate the SD. ANOVA followed by Dunnett’s test was applied for multiple comparisons. *p < 0.05

To further explore the role of thrombin in VM formation, NSCLC cell lines were treated with thrombin in vitro. DTIP and r-hirudin, which are direct thrombin inhibitors, were developed by our group. Tubular network formation by tumor cells in Matrigel was used to detect the ability for VM formation by tumor cells in vitro.^10,11,13,29^ When tumor cells are cultured, the cells elongate and present cellular protrusions. Through the interaction of filaments, cells are arranged in a ring.^30^ With thrombin treatment, A549 and Lewis cells elongated and proliferated in a decentralized manner. Cellular protrusions were more obvious and the cells formed more networks compared with the NS cells. However, with exposure to r-hirudin or DTIP, intercellular connections were damaged and tube structures were reduced or even disappeared (Fig. 2a, b). EMT is the key event underlying VM formation.^31^ E-cadherin, vimentin, N-cadherin, and snail were used as markers to reflect EMT during VM formation.^32^ We detected E-cadherin, N-cadherin, and snail expression in NSCLC cells pretreated with thrombin, r-hirudin, or DTIP. The results indicated that the expression of snail and N-cadherin was reduced, whereas the expression of E-cadherin was increased in both r-hirudin- and DTIP-treated A549 and Lewis cells compared with thrombin-treated cells (Fig. 2c). These results suggested that r-hirudin and DTIP could inhibit the VM formation induced by thrombin in NSCLC cells by regulating EMT.

Thrombin has been reported to activate nuclear factor-κB (NF-κB) signaling in human pleural mesothelial^33^ and SH-SY5Y cells^34^ via PAR-1. The effect of r-hirudin and DTIP on the NF-κB pathway in thrombin-stimulated NSCLC cells was analyzed. The results indicated that thrombin could activate NF-κB signaling in NSCLC cells. Compared with the thrombin-treated group, r-hirudin and DTIP exhibited diminished IκBα and p65 phosphorylation (Fig. 2d), suggesting that r-hirudin and DTIP can inhibit thrombin-induced NF-κB activation.

Studies have shown that NF-κB plays an important role in the EMT process during liver fibrogenesis^35^ and EMT of NSCLC cells occurs via NF-κB activation.^36^ Tian et al.^37^ reported that NF-κB is a master regulator of EMT autocrine loops. We also found that pyrrolidinedithiocarbamate ammonium (PDTC), an inhibitor of NF-κB, could inhibit thrombin-induced increased expression of snail and N-cadherin and inhibit thrombin-induced decreased expression of E-cadherin. lipopolysaccharide (LPS), an activator of NF-κB, could prevent the inhibition induced by r-hirudin and DTIP (Fig. 2e), suggesting that thrombin can regulate EMT via the NF-κB pathway.

### r-hirudin and DTIP inhibit VM formation in a mouse lung cancer model

**Fig. 3:**
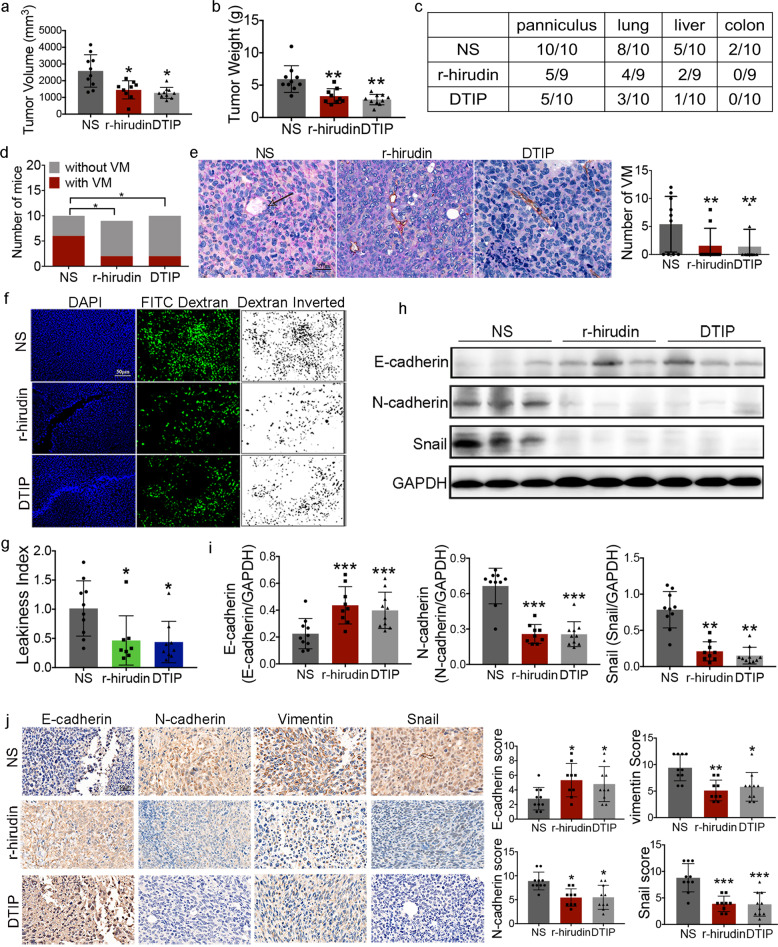
r-hirudin and DTIP inhibited tumor development and VM formation. LLC cells at a density of 1 × 10^6^ in 0.1 mL serum-free media were injected subcutaneously into the right flank of mice. One week after the injection of LLC cells, mice were administered normal saline, 1.0 mg/kg DTIP, or 0.5 mg/kg r-hirudin for 21 consecutive days. **a** Volume of resected tumors derived from the normal saline-, r-hirudin-, and DTIP-treated groups at 45 days after cell injection. **b** Mice were humanely killed and the tumors were resected at 45 days after cell injection. The weights of tumors resected from the normal saline-, r-hirudin-, and DTIP-treated groups. **c** Number of NS-, r-hirudin-, and DTIP-treated mice with panniculus invasion, lung metastasis, liver metastasis, and colon metastasis. **d** Number of NS-, r-hirudin-, and DTIP-treated mice with VM. **e** Representative images of anti-CD31/PAS staining in tumor tissues. Right, the number of VM tubes per tumor tissue sample in different groups. **f** DAPI and FITC-dextran (dextran inverted)-stained tumor sections from resected tumors. **g** Leakiness index of resected tumors. **h** The expression of snail and other EMT markers in tumors was determined by western blotting analysis. **i** Summarized western blot data. **j** Immunohistochemical analysis was performed on tumor samples to determine the expression of E-cadherin, N-cadherin, vimentin, and Snail in tumors from NS-, r-hirudin-, and DTIP-treated mice. Right, scoring of immunostained specimens. All the results are expressed as the mean ± SD. The error bars indicate the SD. Compared with the NS group by one-way ANOVA. *p < 0.05, **p < 0.01, ***p < 0.001

The aforementioned results suggested that thrombin could regulate EMT and promote VM formation in NSCLC cells, and that the novel thrombin inhibitors r-hirudin and DTIP could inhibit thrombin-promoted VM formation. We further confirmed these effects in vivo. Spontaneous metastasis generated through s.c. inoculation of tumors in mice, which involves a comprehensive process, was examined. Daily treatment with 1.0 mg/kg DTIP or 0.5 mg/kg r-hirudin for 21 consecutive days, one week after the injection of LLC cells, was applied. Mice were humanely killed and the tumors were resected 45 days after injection. r-hirudin and DTIP inhibited tumor growth (Fig. 3a, b). Consequently, we counted the number of mice with panniculus invasion and metastases after mice were sacrificed. All mice from the normal saline-treated group showed signs of panniculus invasion, whereas only five of nine mice from the r-hirudin-treated group and five of ten mice from the DTIP-treated group had any noticeable signs of panniculus invasion. Furthermore, the number of mice with lung, liver, and colon metastases was largely reduced in the r-hirudin- or DTIP-treated groups compared with that in the normal saline-treated group (Fig. 3c).

CD31/PAS double staining was performed after tumor tissue was stripped from the mouse model. The results showed that six of ten mice from the normal saline-treated group formed obvious VM structures, whereas in the r-hirudin- and DTIP-treated groups, only two (two of nine) and three (two of ten) mice, respectively, formed VM channels (Fig. 3d); r-hirudin and DTIP reduced the number of VM channels (Fig. 3e). Thrombin and VM have been associated with vascular leakiness.^10,29^ The results revealed that the vessels within normal saline-treated tumors were leakier than those in r-hirudin- or DTIP-treated tumors (Fig. 3f, g). EMT has also been confirmed to be associated with cancer cell invasion and is a key step in VM formation.^38,39^ We further examined the expression of E-cadherin, N-cadherin, vimentin, and snail by western blotting and immunohistochemistry (IHC). r-hirudin and DTIP reduced the expression of N-cadherin, vimentin, and snail, and increased the expression of E-cadherin in primary tumors (Fig. 3h–j). Taken together, the results of vascular leakage analysis, the characteristics of VM, and the results of PAS/CD31 staining suggest that r-hirudin and DTIP could reduce vascular leakage and inhibit EMT, which strengthens the hypothesis that VM is one of the targets of r-hirudin and DTIP in inhibiting tumor development.

Furthermore, we did not find increased bleeding after administration of DTIP. Slight s.c. hemorrhage was observed after r-hirudin administration for 3 weeks continuously (Supplementary Fig. S3a) and no obvious bleeding was found with either r-hirudin or DTIP after 1 week of discontinuation (Supplementary Fig. S3b). These results show that DTIP, a direct thrombin inhibitor, could be applied in anticancer therapy.

### Thrombin expression level with different PAR-1 backgrounds is associated with prognosis of NSCLC patients and formation of VM

**Fig. 4:**
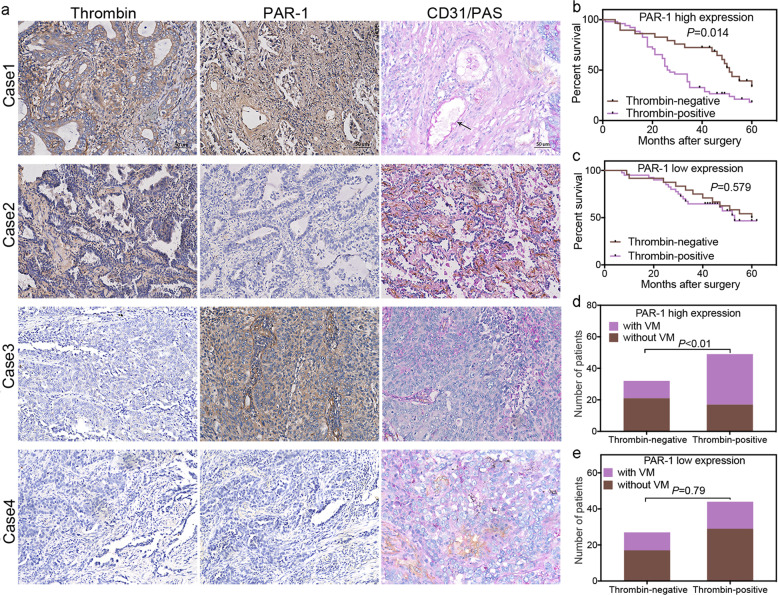
Thrombin with PAR-1 expression is associated with the prognosis of NSCLC patients and the formation of VM. **a** Different expression statuses of thrombin and PAR-1 detected by immunohistochemical staining in consecutive sections of NSCLC tissues from the same patient. The NSCLC tissues of case 1 were positive for thrombin and had high expression of PAR-1; case 2 had tissues positive for thrombin but with low expression of PAR-1; case 3 had tissues negative for thrombin but with high expression of PAR-1; and case 4 had tissues negative for thrombin with low expression of PAR-1. Based on the score of thrombin or PAR-1 in the central positive staining area in the tumor section as a cutoff value, the scores for thrombin and PAR-1 were classified as positive (thrombin+ ≥ 2 and PAR-1 high expression≥6) or negative (thrombin− < 2 and PAR-1 low expression < 6). **b** In Kaplan–Meier analyses, an association of thrombin expression with overall survival (OS), were demonstrated in the PAR-1 high-expression subgroup of NSCLC patients. **c** An association of thrombin expression with overall survival was demonstrated in the PAR-1 low-expression subgroup. **d** An association of thrombin expression with VM formation was demonstrated in the PAR-1 high-expression subgroup. **e** An association of thrombin expression with VM formation was demonstrated in the PAR-1 low-expression subgroup. All the results are expressed as the mean ± SD. The error bars indicate the SD

Thrombin is the main activator of PAR-1.^40^ Our previous experimental results also showed that PAR-1 was highly expressed in NSCLC patients and mice (Supplementary Fig. S4a, f, g), and NSCLC cells (Supplementary Fig. S4h, i). However, we did not find an obvious relationship between the PAR-1 expression levels of tumors and clinical variables, such as the stage of NSCLC differentiation, disease progression, and overall survival (Supplementary Fig. S4a–c). Compared with patients without VM, patients with VM also had a high expression of PAR-1 in lung tumors (Supplementary Fig. S4d), whereas we did not find a consistent relationship between the expression of thrombin and PAR-1 (Supplementary Fig. S4e). Interestingly, we found that in patients with high PAR-1 expression, positive thrombin (thrombin+) expression was associated with a much shorter survival time than negative thrombin (thrombin−) expression (Fig. 4a, b) and patients with thrombin+ expression were more likely to form VM than those with thrombin− expression (Fig. 4a, d). However, no significant difference in the survival and the formation of VM was observed between patients with thrombin+ and thrombin− expression with low expression of PAR-1 (Fig. 4c, e).

### PAR-1 is a major determinant in thrombin-promoted formation of VM in NSCLC

**Fig. 5:**
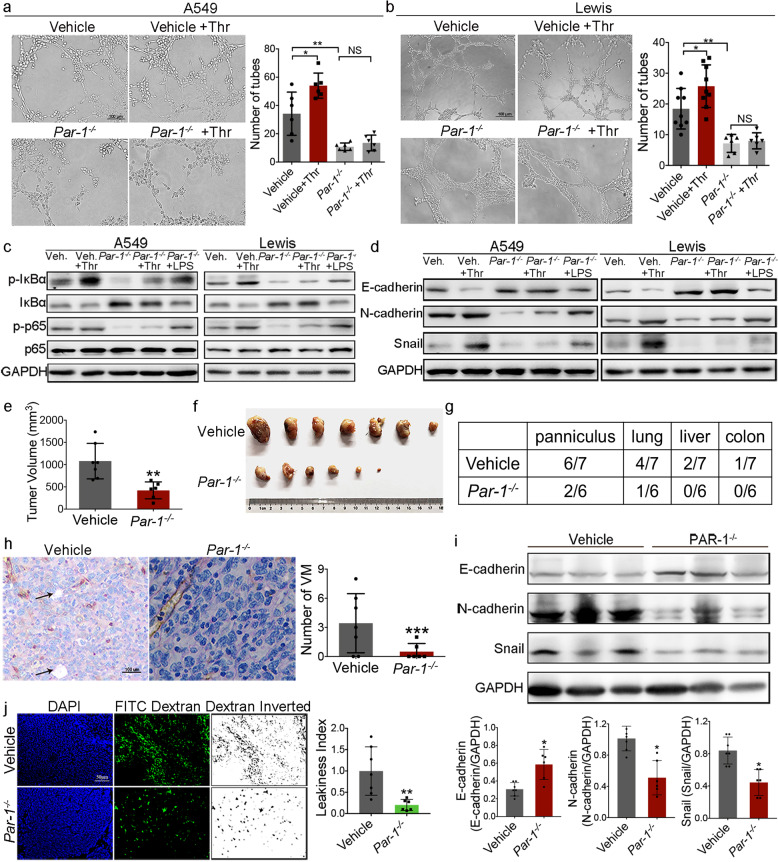
PAR-1 is a major determinant in thrombin-promoted metastasis of lung cancer and formation of VM. **a**, **b** PAR-1 gRNA constructs targeting the PAR-1 gene via lentiviral transduction were used to construct stable A549^*PAR-1−/*^^−^ and LLC^*Par-1−/−*^ cells. An empty construct was also used as a negative control. VM channel formation in PAR-1-deficient A549 and LLC cells. Left, representative photographs of experiments. Right, quantification of the number of VM tubes. **c** Cell lysates were probed for phosphorylated p65, phosphorylated IκBα, and total p65 and IκBα. **d** The expression of snail and other EMT markers in PAR-1-deficient A549 and LLC cells was determined by western blotting analysis. **e**–**j** LLC cells infected with gRNA-PAR-1 lentivirus (*Par-1*^*−/−*^ group) or LV-negative control (NC, vehicle group) were injected subcutaneously into the right flank of mice. **e** Volumes of resected tumors derived from injection of LLC^*Par-1−/−*^ and LLC^NC^ cells into mice at 5 weeks after cell injection. **f** Photograph of tumors resected from each group at 5 weeks after cell injection. **g** Number of mice from different groups with panniculus invasion, lung metastasis, liver metastasis, and colon metastasis. **h** Representative images of anti-CD31/PAS staining in tumor tissues. Right, the number of VM tubes per tumor tissue in the vehicle and *Par-1*^*−/−*^ groups. **i** E-cadherin, N-cadherin, and Snail in resected tumors. The summarized western blot data for EMT markers are given (bottom). **j** DAPI and FITC-dextran (dextran inverted)-stained tumor sections from resected tumors. Right, leakiness index of resected tumors. All the results are expressed as the mean ± SD. The error bars indicate the SD. ANOVA followed by Dunnett’s test was applied for multiple comparisons. *p < 0.05, **p < 0.01, ***p < 0.001, NS, not significant

To observe the role of PAR-1 in thrombin-mediated VM formation more clearly, A549^*PAR-1−/*^^−^ and LLC^*Par-1−/*^^−^ cells were constructed. Our previous studies showed that A549, 95D, PC9, and LLC cells express high levels of PAR-1 compared with normal lung epithelial cells. After PAR-1 knockout, the proliferation of lung cancer cells decreased in vitro. PAR-1 depletion almost completely abrogated thrombin-promoted cell invasion (Supplementary Fig. S5a, b). The VM formation ability of A549^NC^ and A549^*PAR-1−/−*^ cells was also detected in vitro. A549^*PAR-1−/−*^ cells could not form tube-like structures or formed only a few VM-like tubes, and thrombin had no effect on VM-like channels in A549^*PAR-1−/*^^−^ cells (Fig. 5a). Similarly, PAR-1 knockout also reduced the formation of tube-like structures in LLC^*Par-1−/−*^ cells (Fig. 5b). PAR-1 deficiency diminished IκBα and p65 phosphorylation. The ability of thrombin to activate NF-κB was also inhibited (Fig. 5c and Supplementary Fig. S5c, d). After knocking out PAR-1 in A549 and LLC cells, the expression of N-cadherin and snail decreased, whereas that of E-cadherin increased, and thrombin could not rescue the expression level of EMT markers (Fig. 5d and Supplementary Figs. S5e, f and S6). Incubation with LPS rescued the expression level of EMT markers (Fig. 5d and Supplementary Fig. S6e, f). Importantly, thrombin-regulated EMT expression was inhibited by the specific PAR-1 inhibitor ML161 (Supplementary Fig. S6). These results suggested that thrombin could promote EMT and VM formation by PAR-1-mediated NF-κB signaling cascades.

To further analyze the effects of PAR-1 on lung cancer growth, metastasis, and VM formation, we established a lung cancer model in mice using LLC cells infected with gRNA-PAR-1 lentivirus (*Par-1*^*−/−*^ group) or LV-negative control (NC, vehicle group). Mice with s.c. inoculated tumors were injected s.c. with LLC^*Par-1−/−*^ and LLC^NC^ cells and monitored for tumor growth every 3 days. Five weeks after injection of tumor cells, mice injected with control cells had large tumors with an average weight of 1.4 ± 0.4 g and a volume of 884.9 ± 181.4 mm^3^. Interestingly, tumors from the PAR-1-deficient group were smaller than those from the NC group, with an average weight of 0.41 ± 0.10 g and a volume of 178.4 ± 86.7 mm^3^ (Fig. 5e, f). Panniculus invasion, lung metastases, liver metastases, and colon metastases were all largely reduced in the PAR-1-deficient group (Fig. 5g). In addition, VM channel formation was decreased in the PAR-1-deficient tumors (Fig. 5h) and the tumors from the control group were leakier than the PAR-1-deficient tumors (Fig. 5j). The expression of snail and N-cadherin was reduced in tumors with PAR-1 deficiency and E-cadherin expression was increased (Fig. 5i). Together with the in-vitro experiments, these results allowed us to conclude that PAR-1 plays an important role in thrombin-induced VM formation in NSCLC.

### Combination therapy with DTIP and an EGFR inhibitor resulted in improved antitumor efficacy

**Fig. 6:**
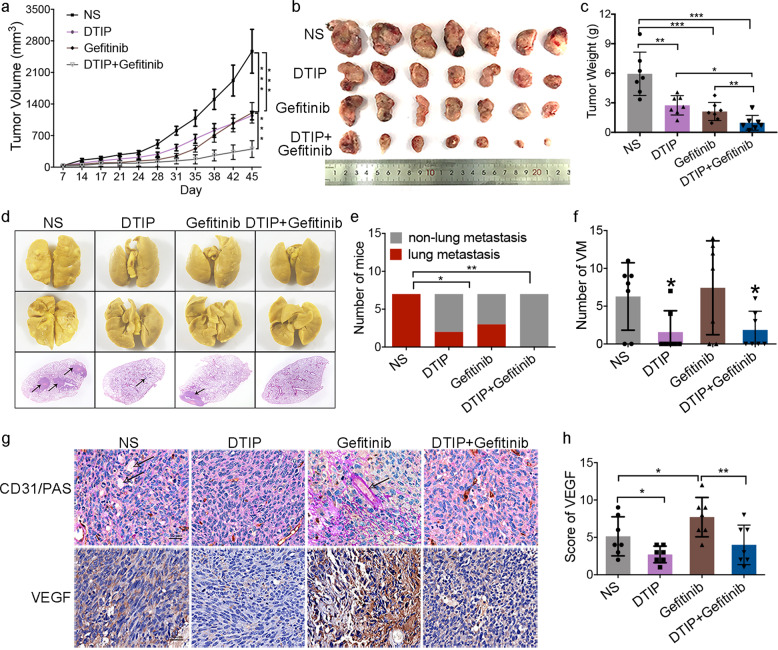
Combination therapy with DTIP and gefitinib results in improved antitumor efficacy. LLC cells (1 × 10^6^) were subcutaneously injected into the right dorsal region of mice. One week after LLC inoculation, the mice were randomly divided into four groups: normal saline, DTIP (1 mg/kg per day, s.c.), gefitinib (100 mg/kg per day, i.g.), and combination DTIP with gefitinib treatment groups, and then the mice were administered DTIP, gefitinib, or normal saline for 21 consecutive days. **a** Tumor growth curve after injection of LLC cells. **b** Mice were humanely killed and the tumors were resected 45 days after cell injection. **c** The weight of resected tumors was determined in different groups. **d** Representative photograph of metastatic nodules in the lungs (upper). H&E-stained sections of representative single lung lobes (bottom). **e** Number of mice with lung metastasis. **f** The number of VM tubes per tumor tissue sample was determined in different groups. **g** Representative images of anti-mouse anti-CD31/PAS staining in tumor tissues (upper). Immunohistochemical analysis was performed to determine the expression of VEGF (bottom). **h** The expression score for VEGF was determined. All the results are expressed as the mean ± SD. The error bars indicate the SD. ANOVA followed by Dunnett’s test was applied for multiple comparisons. *p < 0.05, **p < 0.01, ***p < 0.001, NS, not significant

EGFR-TKIs, including gefitinib, erlotinib, and so on, as angiogenesis inhibitors have significantly improved clinical outcomes. However, most NSCLC patients do not respond (primary resistance) and those who initially respond eventually progress (acquired resistance). There is an urgent need to develop a combination therapy of TKIs with other reagents to enhance antitumor activity and overcome drug resistance. Van der Schaft et al.^41^ reported that angiogenesis inhibitors had no effect on VM and Naumov et al.^42^ reported that EGFR inhibitors could induce a rise in both host and tumor-derived vascular endothelial growth factor (VEGF), which is associated with resistance. One week after LLC inoculation, mice were randomly divided into four groups: normal saline, DTIP (1 mg/kg per day, s.c.), gefitinib (100 mg/kg per day, intragastric), and combination DTIP with gefitinib treatment groups. Next, the mice were administered DTIP, gefitinib, or normal saline for 21 consecutive days. The tumor volume and tumor weight in the combination treatment group were significantly lower than those in the groups administered DTIP alone or gefitinib alone (Fig. 6a–c). The number of mice with lung metastases was largely reduced in the combination treatment group compared with that in the single-treatment groups (Fig. 6d, e). Consistent with previous reports,^41,42^ we found that gefitinib had no effect on VM formation (Fig. 6f, g), and after gefitinib treatment the expression of VEGF was increased (Fig. 6g, h). However, combination therapy with DTIP and gefitinib could inhibit VM formation (Fig. 6f, g), VEGF expression (Fig. 6g, h), and vascular leakage (Supplementary Fig. S7) compared with administration of gefitinib alone. These results indicated that DTIP potentiated gefitinib-induced inhibition of lung cancer and inhibited gefitinib resistance in mice.

## Discussion

In certain circumstances, tumors might have the potential to promote their own growth via alternative metastasis pathways, and metastasis is closely linked to the capacity for VM. VM is related to the expression of markers of pluripotent stem cells, invasive disease, and poor prognosis of patients, and VM is also related to tumor spread and metastasis.^11^ We found that there were VM tubes in tumor samples of NSCLC patients and a high VM ratio was associated with poor prognosis. This result is consistent with the hypothesis that VM occurs through aggressive tumor “stem-like” phenotypes,^43^ leading to worse clinical outcomes. Patients who survived >5 years had low VM ratios. VM formation may be a biomarker for tumor prognosis. VM may be a potential therapeutic target. Prospective studies will be helpful to explore the molecular regulatory mechanism of VM.

It has long been presumed that tumors may take advantage of the hemostatic system. There are many significant hemostatic abnormalities in cancer patients. In fact, hemostasis complications are a common cause of death in cancer patients. Studies have shown that exogenous thrombin is capable of enhancing tumor adhesion to platelets,^44^ endothelial cells,^45^ and fibronectin^46^ in vitro and revealed that exogenous thrombin promotes tumor growth.^47^ In our study, we found that thrombin was overexpressed in NSCLC, and that the level of thrombin was correlated with poor prognosis of NSCLC patients, which might be a reason why malignant tumors often exhibit hypercoagulability. The mechanism by which thrombin expression is increased in NSCLC needs to be further studied in the future. Interestingly, we also found that the expression of thrombin in tumors is closely related to the formation of VM. The expression of thrombin in tumors was increased in patients with VM. Hence, we hypothesize that VM might be driven by increased expression of thrombin.

To further study the effect of thrombin on VM formation, NSCLC cell lines were treated with thrombin in vitro. The results showed that 10 nmol/L thrombin could promote NSCLC cell elongation and proliferation to varying degrees. It also promoted VM tube formation. r-hirudin and DTIP are new bivalent direct thrombin inhibitors that could effectively prevent the formation of thrombosis and embolism.^26,27^ However, in vitro, r-hirudin and DTIP were effective against thrombin-induced tubular network formation in lung cancer cells. In lung cancer models, r-hirudin and DTIP inhibited tumor progression and spontaneous metastasis, and in r-hirudin- or DTIP-treated mice, VM formation was likely inhibited compared with that in normal saline-treated mice. This suggests that VM might be a target of r-hirudin and DTIP in suppressing tumor progression.

A high degree of tumor leakiness has been associated with VM, in which tumor cells differentiate into endothelial-like cells and form extracellular matrix-rich tubular structures to carry blood from the vasculature to hypoxic regions of the tumor.^10,29^ Vascular leakage^10,29^ and EMT^11,38,39^ are two important characteristics of VM formation. Furthermore, r-hirudin and DTIP inhibited EMT in vitro and in vivo, and reduced vascular leakage in mouse tumor models. Taken together, the results of vascular leakage analysis, the characteristics of VM, and the results of PAS/CD31 staining suggest that r-hirudin and DTIP could reduce vascular leakage and inhibit EMT, which strengthens the hypothesis that VM is one of the targets of r-hirudin and DTIP in inhibiting tumor development.

It was reported that thrombin, acting through PAR-1, promotes EMT in embryo development.^25^ Our studies have found that PAR-1 is a major determinant in thrombin-promoted metastasis of lung cancer; however, the contribution of thrombin/PAR-1 to EMT and VM formation is still unknown. Our previous studies suggested that PAR-1 was highly expressed in human NSCLC tissues but showed no difference based on subtype and clinical stage. Interestingly, we found that the relationships between thrombin and NSCLC prognosis, and VM formation were different in patients with various PAR-1 expression levels. Thrombin expression was closely associated with VM formation and survival in NSCLC patients with higher PAR-1 levels; however, it was not significantly related with VM formation and survival in NSCLC patients with low PAR-1 expression. NSCLC patients with thrombin-positive/PAR-1-high expression had the poorest prognosis and were the most likely to form VM. These findings suggest that thrombin, together with PAR-1, substantially contributes to VM formation and NSCLC malignancy.

To further investigate the effect of thrombin on PAR-1-mediated VM formation, PAR-1-deficient NSCLC cells were treated with thrombin in vitro. We found that the ability of PAR-1-deficient NSCLC cells to form VM tubes was lost; thrombin had no effect on their VM formation capacity.

The cleavage of PAR-1 by thrombin leads to the activation of NF-κB signaling cascades.^33^ Studies have shown that NF-κB plays an important role in the EMT process. In this study, we found that thrombin could activate NF-κB signaling and induce EMT, and that r-hirudin and DTIP could inhibit thrombin-induced NF-κB activation and EMT. PDTC, an inhibitor of NF-κB, could inhibit thrombin-induced EMT. Thrombin is the main activator of PAR-1. In PAR-1-deficient NSCLC cells, thrombin had no effect on NF-κB activation, EMT, and VM formation, whereas LPS could rescue NF-κB activation and EMT. These results suggested that thrombin promoted the activation of NF-κB through the activation of PAR-1, thereby inducing EMT and VM formation. r-hirudin and DTIP could bind to thrombin specifically and block thrombin binding to PAR-1, thereby inhibiting NF-κB activation and reducing the expression of N-cadherin and snail, while increasing the expression of E-cadherin in NSCLC cells, and this mechanism is very beneficial for suppressing VM formation. This study is the first to illustrate the effect of thrombin inhibitors on the formation of VM.

A series of studies in the early 2000s observed that distinct epidemiological subgroups of patients with NSCLC had dramatically enhanced responses to treatment with the ATP-competitive, reversible EGFR inhibitors gefitinib and erlotinib.^48^ Meanwhile, EGFR inhibitors as angiogenesis inhibitors are significantly associated with an increased risk of arterial thromboembolism.^49^ Less than 10% of NSCLC patients have an objective tumor response. In addition, it is an interesting possibility that antiangiogenic therapy may lead to a selective growth advantage favoring VM, thus leading to drug-induced resistance.^12^ Based on the findings of this study, we conclude that the highly specific, potent thrombin inhibitor DTIP inhibits VM formation in NSCLC. In our studies, we found that gefitinib, an EGFR inhibitor, did not inhibit VM formation. Similar results have been reported by Rybak et al.^50^ and Van der Schaft et al.^41^ Gefitinib could increase the expression of VEGF, which is consistent with the results reported by Naumov et al.^42^ These results might be associated with gefitinib resistance. Interestingly, combination therapy with DTIP with gefitinib could inhibit VM formation and VEGF expression. DTIP potentiated gefitinib-induced inhibition of lung cancer and inhibited gefitinib resistance in mice. Combination therapy with DTIP with gefitinib resulted in improved antitumor efficacy. This approach merits further evaluation in more extensive studies and in NSCLC patients.

In this study, we not only showed that thrombin plays a crucial role in VM but also explained why hypercoagulability promotes cancer progression. VM formation may be a biomarker for tumor prognosis. We conclude that thrombin plays an important role in PAR-1-mediated VM formation via NF-κB signaling. We suggest that treatment with DTIP should start immediately after diagnosis (before extensive tumor development) and should be administered in conjunction with EGFR inhibitors. Our future experiments will focus on the mechanisms, the reason why the expression of thrombin is increased in NSCLC, and the ability of combination therapy with thrombin inhibitors and angiogenesis inhibitors to achieve a better therapeutic effect.

## Materials and methods

### Materials

The expression and purification of r-hirudin and DTIP have been described previously.^26,27^ Detailed materials are described in the Supplementary Materials.

### Animals

C57BL/6 mice (5–6 weeks, male) were purchased from Shanghai SLAC Laboratory Animal Limited Company. All animal procedures were carried out in accordance with institutional guidelines at Fudan University.

### Patients and tissue slides

All experiments using human tissue slides were performed in accordance with the Declaration of Helsinki and approved by the Institutional Review Board of Fudan University. The clinical samples of lung cancer patients were obtained from Fudan University Shanghai Cancer Center (Shanghai, China). The tissue slides consisted of samples from 152 patients with NSCLC, including 118 cases of adenocarcinoma, 25 cases of squamous cell carcinoma, and 9 cases of other types of NSCLC at different stages. Healthy lung tissues from each sample were also included. The clinicopathological features of 152 NSCLC tissues are shown in Supplementary Table 1.

### Vasculogenic mimicry formation assay

Ninety-six-well plates were precoated with 50 μL liquid Matrigel per well. After incubation at 37 °C for 1 h, A549 cells (1.5 × 10^4^ cells/well) or Lewis cells (0.5 × 10^4^ cells/well) suspended in RPMI-1640 medium with phosphate-buffered saline, 10 nmol/L thrombin, 25 nmol/L r-hirudin, 50 nmol/L DTIP, 10 nmol/L thrombin + 25 nmol/L r-hirudin, or 10 nmol/L thrombin + 50 nmol/L DTIP were seeded and cultured for 4 h. The cells in five randomly chosen fields were photographed and counted. VM formation as measured by the capacity to form loops was quantified by AngioSys 2.0 (TCS Cellworks, Buckingham, England) and the number of loops was regarded as the number of tubes.

### Immunohistochemistry and scoring of immunostained specimens

Immunostained specimens were reviewed by two pathologists blinded to the patients’ clinical status. To evaluate the expression of proteins using IHC, the intensity of protein staining was graded by consensus on a scale from 0 to 3 (0 was negative staining; 1 was weakly positive staining; 2 was moderately positive staining; and 3 was strongly positive staining). The frequency of positive cells was graded by consensus on a scale from 0 to 4 (0 was positivity in <1% of cells; 1 was positivity in 1–10% of cells; 2 was positivity in 10–50% of cells; 3 was positivity in 50–80% of cells, and 4 was positivity in >80% of cells). The immunohistochemical scores were determined by the staining intensity and the frequency of positive cells (staining intensity score × positive cell score). Based on the score of thrombin or PAR-1 staining in the central positive staining area in the tumor section as a cutoff value, the score (staining intensity score × positive cell score) of thrombin or PAR-1 was classified as positive (thrombin+ ≥ 2 and PAR-1 high expression ≥ 6) or negative (thrombin− < 2 and PAR-1 low expression < 6).

### VM detection

PAS staining and CD31 IHC were used to evaluate the presence and extent of mimicry as previously described.^11,14,29^ Each sample was scored for the number and size of areas with morphology consistent with mimicry. The criteria used were as follows: (1) the presence of PAS-positive channels that contained red cells and fluid, (2) the absence of CD31 staining in these channels, and (3) the polarization of tumor cells on an indistinct or imperceptible matrix lining vascular channels with red cells and/or fluid and no evidence of endothelization or tumor cells lining vascular spaces with no evidence of a matrix. After PAS and CD31 staining, the whole sections were observed via slice digital scanning (3DHISTECH, Ltd). If typical VM structures could not be found in the whole slice, the sample was considered to be from a “patient without VM.”

### Subcutaneous inoculation of tumors in mice

This animal study was conducted in accordance with the rules and regulations of the Institutional Animal Care and Use Committee at the Department of Laboratory Animal Science, Fudan University (Shanghai, China). Tumor cells at a density of 1 × 10^6^ in 0.1 mL serum-free media were injected s.c. into the right flank of mice. The mice were monitored daily for the development of visible tumors. Once a tumor was clearly visible, it was calipered three times each week, and the volume was estimated using the formula *V* = (*LW*^2^)/2, where *V* is the volume, *L* is the longest diameter, and *W* is the shortest diameter. After mice were killed, the tumors were removed; some were fixed in 4% paraformaldehyde (PFA) and the other tumors were lysed for western blotting.

Paraffin sections were made from the largest section of mouse tumor tissues and were used for PAS and CD31 staining. After PAS and CD31 staining, we observed the whole section via slice digital scanning (3DHISTECH, Ltd). The number of typical VM channels in the whole tumor tissue section of each mouse was counted. If there were no typical VM channels in the whole tumor tissue section, the number of VM components was regarded as 0.

### Vascular leakage

Mice were injected with 0.1 mL fluorescein isothiocyanate dextran (10 mg/mL, 20 kDa, Sigma Aldrich) by tail vein injection. After 30 min, the mice were anesthetized and perfused with 4% PFA. After fixation, tumors were collected and placed in 4% PFA overnight at 4 °C. After this, samples were infiltrated with 20% sucrose overnight at 4 °C. Tumors were frozen in OCT compound (Sakura Finetek) and 15 μm-thick sections were cut, washed, incubated with 4′,6-diamidino-2-phenylindole, and mounted in ProLong Gold antifade reagent (Life Technologies). Sections were examined under a fluorescence microscope. An average of five fields were analyzed from each sample. Images were quantified using ImageJ software.

### Statistical analysis

All data are expressed as the mean ± SD. Unless otherwise stated, differences between the two groups were analyzed by unpaired *t*-test when variances were equal and one-way analysis of variance followed by the Newman–Keuls test was used for multiple comparisons with Prism 6 (GraphPad, Inc.). *P* < 0.05 was considered to be statistically significant.

## Supplementary information

Supplementary information
