# Supplementary material for: Thrombin is a therapeutic target for non-small-cell lung cancer to inhibit vasculogenic mimicry formation
Source: Signal Transduct Target Ther. 2020 Jul 10;5:117. doi: 10.1038/s41392-020-0167-1 (PMC7347850; doi:10.1038/s41392-020-0167-1)
Supplement: Supplementary file 1 — Supplementary information [file 41392_2020_167_MOESM1_ESM.docx]

Supplementary Materials for

Thrombin is a therapeutic target for non-small cell lung cancer to inhibit vasculogenic mimicry formation

Zhao Bing^1, 2^, Wu Mengfang^1, 2^, Hu Zhihuang^4, 5^, Ma Yixin^1, 2^, Wang Qi^1, 2^, Zhang Yanling^1, 2^, Li Yaran^1, 2^, Yu Min^1, 2^, Wang Huijie^4, 5*^, Mo Wei^1, 2, 3*^

Correspondence to: [15111010026@fudan.edu.cn](mailto:15111010026@fudan.edu.cn)

**This PDF file includes:**

Materials and Methods

Figures. S1 to S7

Table S1

**Materials and Methods**

Materials

The antibody specific for thrombin, PAR-1, VEGF, and CD31 was from Santa Cruz (Santa Cruz Biotechnology, Santa Cruz, CA). The anti-GAPDH, anti-histone H3, anti-E-cadherin, anti-N-cadherin, and anti-snail antibodies were from Proteintech (Proteintech Group, Chicago, IL). The anti-NF-κB p65, anti-phospho-NF-κB p65, anti-IκBα and anti-phospho-IκBα were purchased from Cell Signaling Technology (Danvers, MA). Thrombin and LPA were obtained from Sigma Aldrich (St Louis, MO). ML161 (PAR-1 antagonist), PDTC and gefitinib were purchased from TargetMol (Boston, USA).

**Cell culture**

A549, 95D, PC9 and Lewis cells (NSCLC cell of mice) were maintained in RPMI-1640 medium supplemented with 10% fetal bovine serum (FBS) and 1% penicillin/streptomycin, 293T were maintained in DMEM with 10% FBS and 1% penicillin/streptomycin. The cells were cultured at 37^°^C and 5% CO2 in a humidiﬁed incubator.

**Cell** **invasion**

Cell invasion was measured using transwell inserts (Corning Life Sciences, Tewksbury, MA) according to manufacturer’s instructions. Briefly, transwell chambers (8 mm pore size) were coated with 50 μL of diluted matrigel. Cells suspended in serum-free medium at a density of 1.5×10^5^ cells/mL were seeded (0.1 mL) in the upper chambers and 0.5 mL medium containing 10% FBS was added to the lower chambers. After culturing for 24 hours, cells were ﬁxed in methanol and stained with 0.1% crystal violet. The cells on the bottom of the filters were counted in three random microscope fields.

**gRNA design and lentivirus infection**

CRSIPR guides targeting *PAR-1* of human (GAAGGTCAAGAAGCCGGCGG) and mouse sequences (GCGACGATCAGCAAGCGCCG) were generated and cloned into LentiCRISPRv2 at BsmBI restriction sites. LentiCRISPRv2 and packing constructs were transfected into 293T cells. Virus supernatants were collected 48 h after transfection. A549 and LLC cells were infected with viral supernatants in the presence of polybrene (8 μg/ml) and were then selected in growth media containing 2-10 μg/ml puromycin. All the cell lines used have been tested and authenticated by karyotyping. Transfection with the empty plasmid was performed which was used as negative control (NC).

**Western blotting**

Total proteins were extracted using an extraction buffer with a protease inhibitor cocktail (CWBIO). Proteins was separated by SDS-PAGE and then transferred to Immobilon-P transfer membrane (Millipore Corp). The membranes were blocked with 5% (w/v) non-fat dry milk for 1 h at room temperature, followed with overnight incubation at 4^°^C with primary antibodies. Immunoreactive proteins were detected using ECL Plus, after secondary antibody incubation.

**Bleeding assay**

A transverse incision was made with a scalpel over a lateral tail vein at a position where the distal end of the tail is 5.0 mm of mice. Following the incision, the tail was immediately immersed in 0.9% sodium chloride kept at 37°C in a 50 mL Corning tube. The test tube was gently rotated or moved up and down to prevent the shed blood from obscuring the incision site. The time from the incision to the cessation of bleeding was recorded as the bleeding time. The occurrence of bleeding side effects of r-hirudin and DTIP was determined after the last administration of the r-hirudin and DTIP and one week after the last dose.

**Figure. S1.**

**
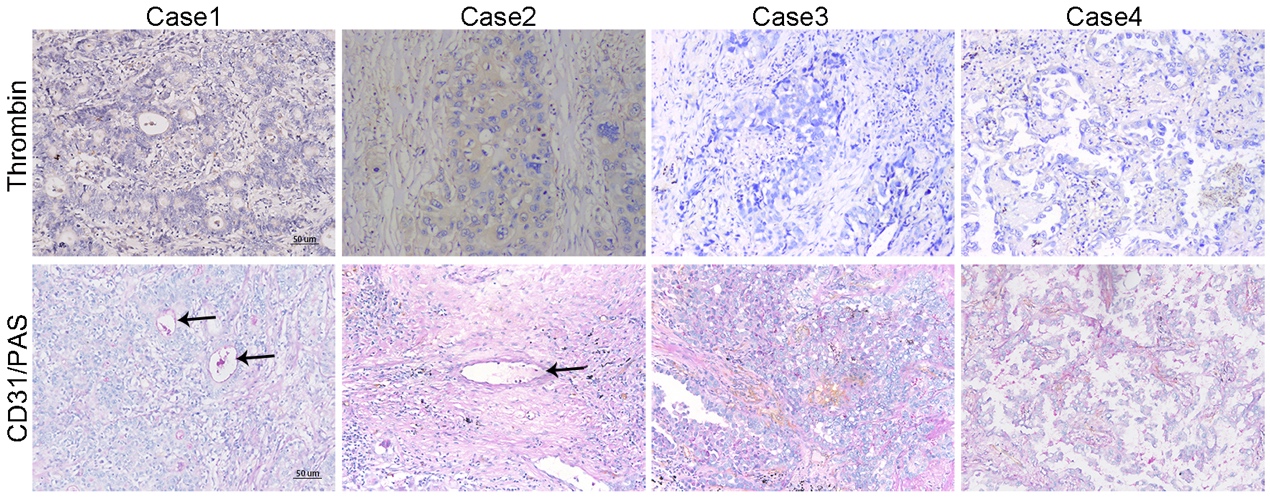
**

**Supplementary Fig. 1 Thrombin expression is related to the formation of VM in NSCLC patients.** Region showing typical malignant morphology of the NSCLC and thrombin expression in corresponding tissues, PAS+/CD31- VM tubes (black arrow). Case 1 and case 2 are positive for thrombin and VM; case 3 and case 4 are negative for thrombin and VM.

Figure. S2.

**
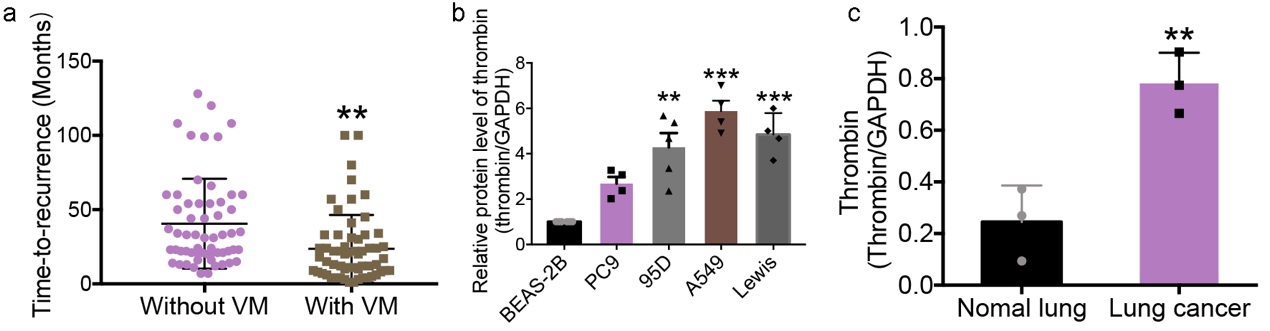
**

**Supplementary Fig. 2 The formation of VM in NSCLC is closely related to the prognosis of patients and thrombin is overexpressed in NSCLC cells. a** Time-to-recurrence in NSCLC patients with or without VM. **b** Summary data of the expression of thrombin in BEAS-2B, A549, 95D, PC9 and Lewis cells that was determined by western blotting analysis. **c** Summary data of the expression of thrombin in tumor tissues of mice and normal lung tissues of mice that was determined by western blotting analysis. All the results were expressed as mean ± SD. ANOVA followed by Dunnett's test was applied for multiple comparison. **p* < 0.05, ***p* < 0.01, ****p* < 0.001.

**Figure. S3.**


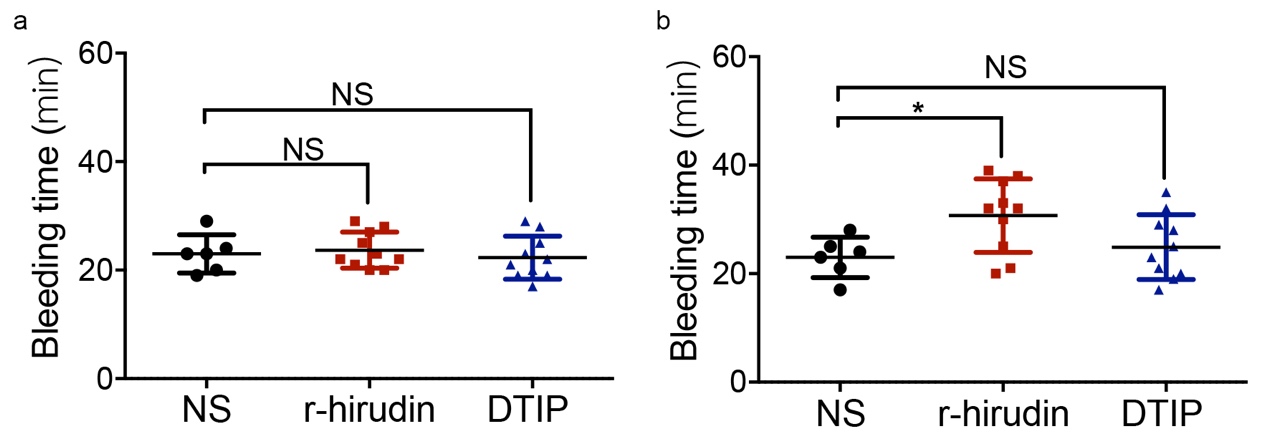


**Supplementary Fig. 3 Bleeding effect of r-hirudin and DTIP in mice.** The distal 5 mm of the mice tail was transected to determine the occurrence of bleeding side effects of r-hirudin and DTIP after the last administration of the r-hirudin and DTIP and one week after the last dose. (A) Bleeding time was determined after the last administration of r-hirudin and DTIP for three weeks continuously. (B) Bleeding time was determined one week after the last dose by measuring bleeding time after tail snip. Data are expressed as mean ± SD. Compared with NS group by one-way ANOVA. **p* < 0.05, ***p* < 0.01, NS, not significant.

**Figure. S4.**


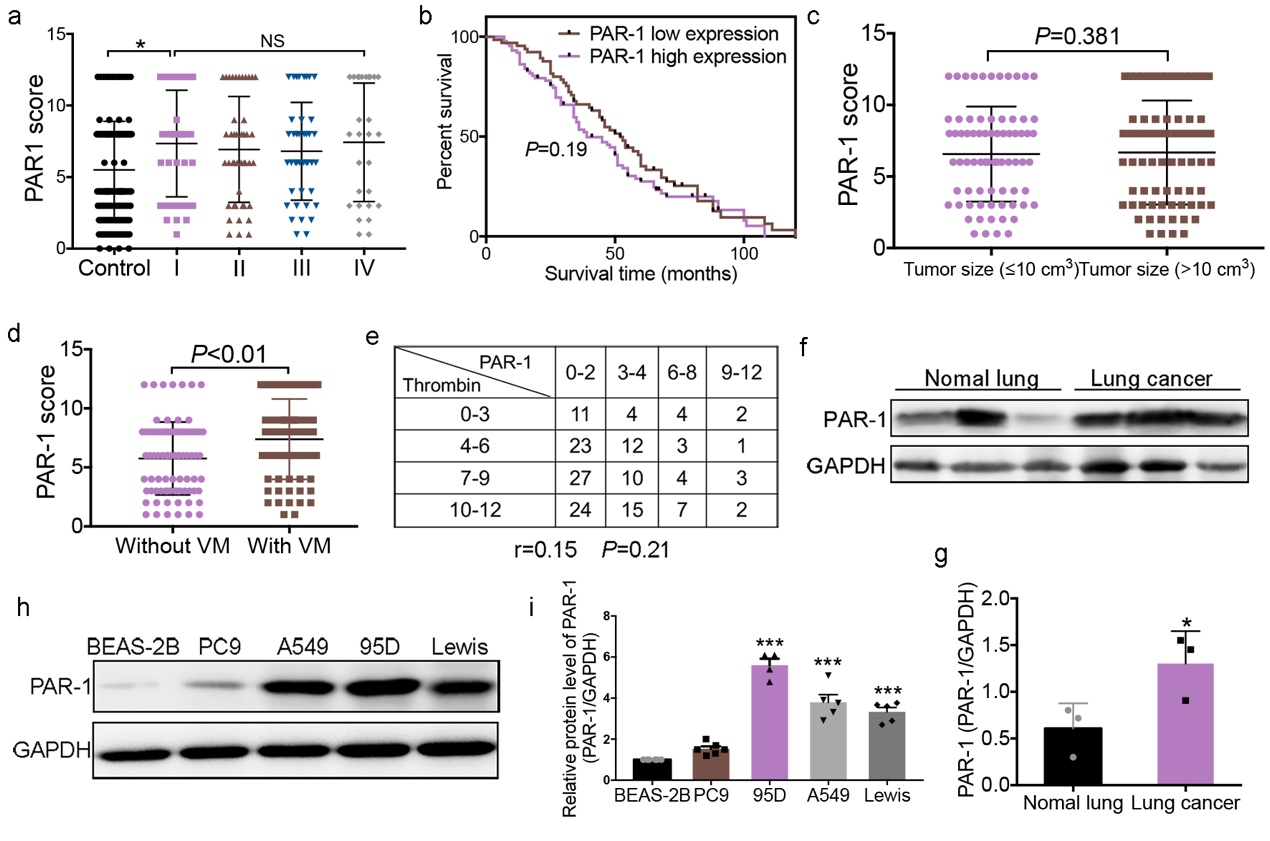


**Supplementary Fig. 4 PAR-1 expression in NSCLC patients and NSCLC cells. a** PAR-1 expression in adjacent non-tumor lung tissue and in in different stages of NSCLCs. **b** Univariate survival analysis according to PAR-1 expression in NSCLC patients, Kaplan–Meier survival analysis for patients dichotomised by PAR-1 low or high expression (PAR-1 high expression ≥ 6, PAR-1 low expression <6, 6 is immunohistochemical scores which is determined by staining intensity grade × positive cell grade). **c** The association of PAR-1 expression with tumor size of NSCLC patients. **d** The relationship between expression level of PAR-1 and the formation of VM in tumor tissues. **e** The association of thrombin expression and PAR-1 expression in NSCLC patients. **f** The expression of PAR-1 in tumor tissues of mice and normal lung tissues of mice was determined by western blotting. **g** Summary data of western blotting were given**. h** The expression of PAR-1 in 95D, PC9, A549 and Lewis cells was determined by western blotting analysis. **i** Summary data of western blotting were given. All the results were expressed as mean ± SD. ANOVA followed by Dunnett's test was applied for multiple comparison. **p* < 0.05, ***p* < 0.01, ****p* < 0.001. NS, not significant.

**Figure. S5.**


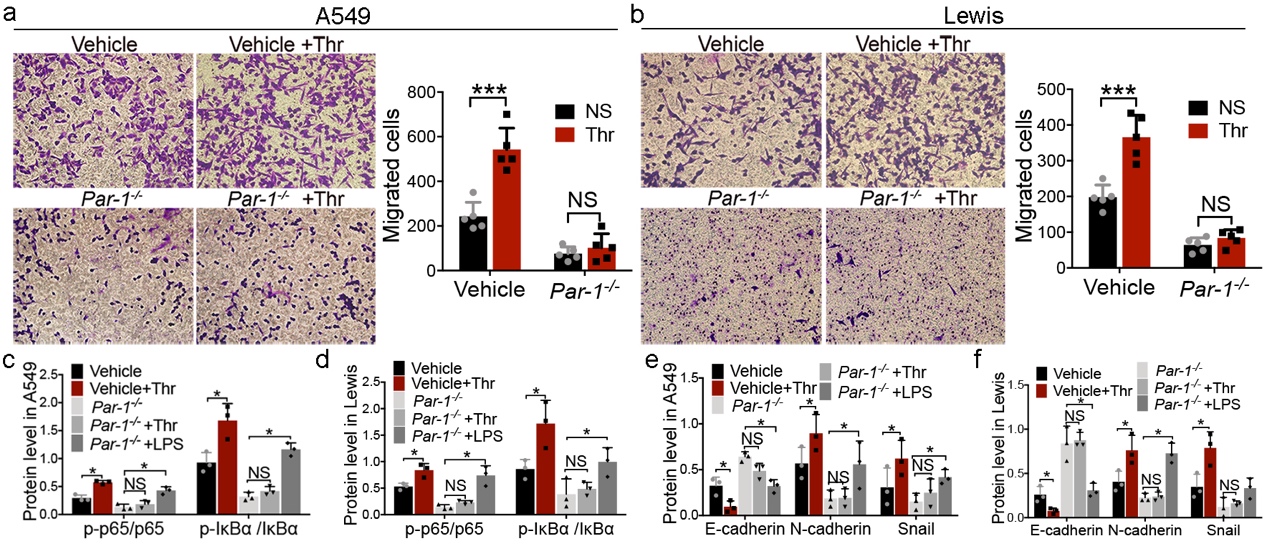


**Supplementary Fig. 5 PAR-1 is a major determinant in thrombin-promoted formation of VM in NSCLC. a** Representative picture of A549 cells migrated through the transwell are shown, Right, quantitative analysis of invasive cells. **b** Representative pictures of LLC cells migrated through the transwell are shown, Right, quantitative analysis of invasive cells. **c** Summary data of western blotting of phosphorylated p65, phosphorylated IκBα in A549 cells. **d** Summary data of western blotting of phosphorylated p65, phosphorylated IκBα in LLC cells. **e** Summary data of western blotting of E-cadherin, N-cadherin, and Snail in A549 cells. **d** Summary data of western blotting of E-cadherin, N-cadherin, and Snail in LLC cells. All the results were expressed as mean ± SD. ANOVA followed by Dunnett's test was applied for multiple comparison. **p* < 0.05, ***p* < 0.01, ****p* < 0.001. NS, not significant.

**Figure. S6.**


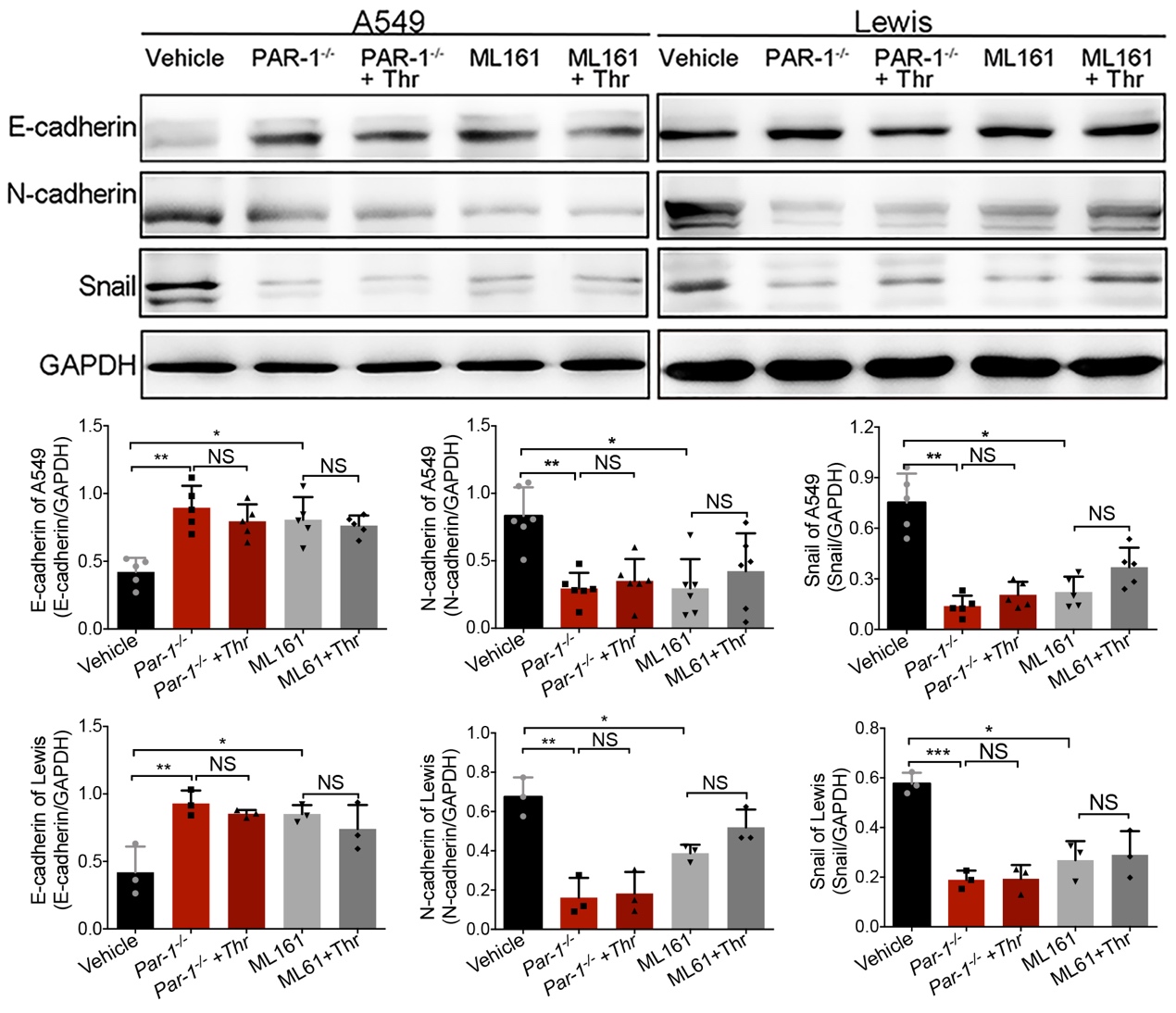


**Supplementary Fig. 6 Thrombin promote EMT via PAR-1 signal in vitro.** The expression of snail and other EMT markers in PAR-1 deficient A549 and LLC cells was determined by western blotting analysis. Bottom, Summary data of western blotting of E-cadherin, N-cadherin, and Snail in A549 and LLC cells. All the results were expressed as mean ± SD. ANOVA followed by Dunnett's test was applied for multiple comparison.

**Figure. S7.**

**
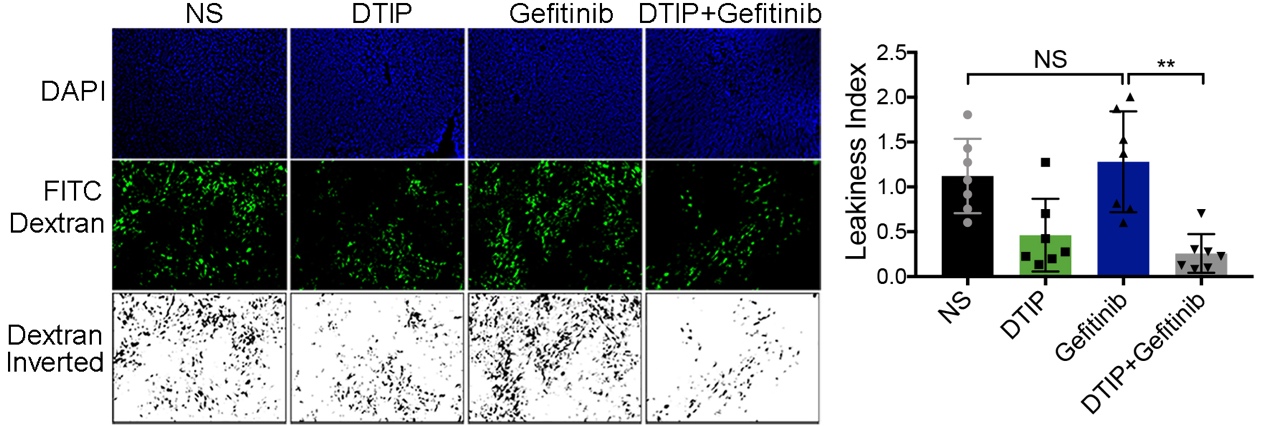
**

**Supplementary Fig. 7 Combination therapy of DTIP and** **gefitinib results in improved anti-tumor efficacy.** DAPI and FITC dextran (dextran inverted)-stained tumor sections from resected tumors. Right, leakiness index of resected tumors. All the results were expressed as mean ± SD. ANOVA followed by Dunnett's test was applied for multiple comparison. ***p* < 0.01; NS, not significant.

Table S1.

| **Supplementary Table 1.**  Clinicopathologic data of 152 NSCLC patients | | |
| --- | --- | --- |
| Characteristic | Patient | |
|  | N | % |
| Sex  Male  Female | 82  70 | 53.9  46.1 |
| Age, Median (58)  ≤ 58  < 58 | 66  86 | 43.4  56.6 |
| Tumor size (cm)  D ≤ 2  2 < D ≤ 5  D > 5 | 57  69  26 | 37.5  45.4  17.1 |
| TNM stage  I  II  III  IV | 44  38  41  29 | 28.9  25.0  27.0  19.1 |
| Lymphatic metastasis  No  Yes | 58  94 | 38.2  61.8 |
| Differentiation  I  II  III | 36  62  52 | 23.7  40.8  34.2 |
